# G-Quadruplexes as Sensing Probes

**DOI:** 10.3390/molecules181214760

**Published:** 2013-11-28

**Authors:** Branislav Ruttkay-Nedecky, Jiri Kudr, Lukas Nejdl, Darina Maskova, Rene Kizek, Vojtech Adam

**Affiliations:** 1 Department of Chemistry and Biochemistry, Faculty of Agronomy, Mendel University in Brno, Zemedelska 1, Brno CZ-613 00, Czech Republic; E-Mails: brano.ruttkay@seznam.cz (B.R.-N.); george.kudr@centrum.cz (J.K.); lukasnejdl@gmail.com (L.N.); kizek@sci.muni.cz (R.K.); 2 Central European Institute of Technology, Brno University of Technology, Technicka 3058/10, Brno CZ-616 00, Czech Republic; E-Mail: darina.maskova@seznam.cz

**Keywords:** G-quadruplex, DNAzyme, hemin

## Abstract

Guanine-rich sequences of DNA are able to create tetrastranded structures known as G-quadruplexes; they are formed by the stacking of planar G-quartets composed of four guanines paired by Hoogsteen hydrogen bonding. G-quadruplexes act as ligands for metal ions and aptamers for various molecules. Interestingly, the G-quadruplexes form a complex with anionic porphyrin hemin and exhibit peroxidase-like activity. This review focuses on overview of sensing techniques based on G-quadruplex complexes with anionic porphyrins for detection of various analytes, including metal ions such as K^+^, Ca^2+^, Ag^+^, Hg^2+^, Cu^2+^, Pb^2+^, Sr^2+^, organic molecules, nucleic acids, and proteins. Principles of G-quadruplex-based detection methods involve DNA conformational change caused by the presence of analyte which leads to a decrease or an increase in peroxidase activity, fluorescence, or electrochemical signal of the used probe. The advantages of various detection techniques are also discussed.

## 1. Introduction

DNA plays a fundamental role in all living organisms, as it is a crucial molecule responsible for the storage and copying of genetic information [1]. Previously, it has been assumed that DNA has a “passive” structure used only for the storage of genetic information. From the experiments carried out recently, it is evident that DNA is a very dynamic molecule, capable of forming a number of spatial arrangements. These structures include single-stranded hairpins, homoduplexes, triplexes, and quadruplexes [2]. Formerly these structures were considered an interesting phenomenon with a little practical meaning. Later it was found that the formation of these structures takes place under certain physiological conditions; therefore their involvement in recombination, regulation of gene expression and proliferation of tumour cells is assumed. Based on these facts it is not surprising that there is growing interest in the structures of nucleic acids as potential therapeutic drugs [3].

### G-Quadruplexes

**Figure 1:**
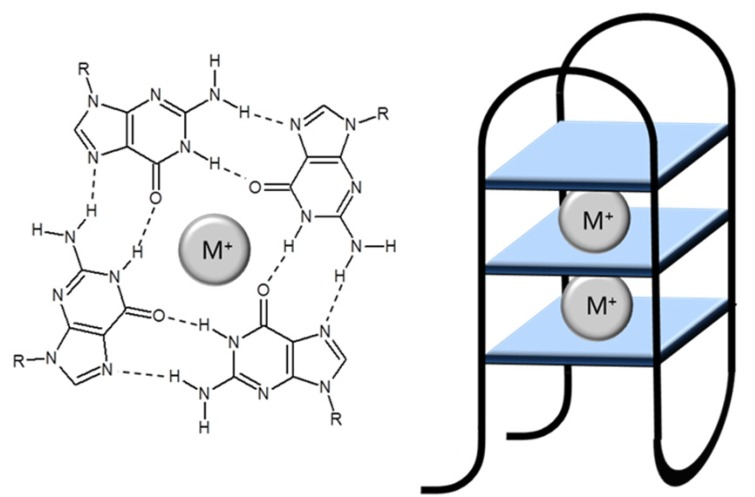
Left: structure of a G-quartet, with four guanines arranged around a central monovalent cation (M^+^). Right: structure of a G-quadruplex, in this example an antiparallel unimolecular structure with three stacked quartets. Adopted and modified according to Huppert *et al.* [4].

The most famous structures of DNA are highly ordered guanine quadruplexes (G-quadruplexes) composed of guanine quartets (G-quartets), which are formed from four guanine bases. In G-quartet each guanine is linked with neighbouring guanine via two hydrogen bonds by Hoogsteen pairing. These structures then stack on each other in a helical fashion, forming a G-quadruplex structure (Figure 1).

G-quadruplexes are stabilized by hydrogen bonds and by the presence of alkali metal ions, which are located in the centre between two G-quartets. These ions are most frequently potassium or sodium cations, which are connected by electrostatic interactions on the guanine carbonyl [4,5,6,7,8]. G-quadruplexes are characterized by unique architecture and high stability [9]. Some sequences remain folded under physiological conditions and at temperatures above 90 °C [4].

G-quadruplexes are highly polymorphic and they can be classified in terms of the stoichiometric as unimolecular, bimolecular, and tetramolecular and also in terms of orientation as parallel, antiparallel or mixed. Structure of G-quadruplexes depends on the composition and length of the DNA, on the orientation of the chains and positions of the loops, and also on the nature of the cations [10]. Due to these modifications G-quadruplex structures can be created easily both intermolecularly and intramolecularly [1].

G-quadruplex structures have drawn the attention of researchers in medicinal chemistry, supramolecular chemistry, and nanotechnology [6,11,12,13]. In addition, G-quadruplexes have been used as basic units in the formation of nanostructures [12]. Almost all G-quadruplex structures studied have been formed by one, two, or four G-rich strands [6]. G-quadruplex structures formed by three strands, leading to a tri-G-quadruplex species have been described recently by Zhou *et al.* [14]. This tri-G-quadruplex design may also provide a new avenue for creating nanoscale materials.

Human telomeric DNA composed of (TTAGGG/CCCTAA)n repeats may form a classical Watson-Crick double helix. Each individual strand is also prone to quadruplex formation: the G-rich strand may adopt a G-quadruplex conformation involving G-quartets whereas the C-rich strand may fold into an i-motif based on intercalated C.C^+^ base pairs [15]. A number of research groups constructed different nanodevices based on switching between structures as induced by changes in environmental factors [16,17,18]. Zhou *et al.* [19] demonstrated the coexistence of a G-quadruplex and an i-motif in a single strand. This structure was built on the basis of the principle that G-quadruplex formation requires the presence of a G-quadruplex-compatible cation, whereas i-motif formation demands acidic conditions. The constructed nanodevice is very simple and can be rapidly converted into other structures by varying the stimulus, such as the pH value or cation.

G-quadruplexes form a complex with hemin (G-quadruplex/hemin) and are called DNAzymes. These complexes exhibit peroxidase-like activity and effectively catalyse the H_2_O_2_-mediated oxidation of 2,2'-azino-bis(3-ethylbenzothiazolin-6-sulfonic acid)diammonium salt (ABTS) [20,21,22]. Due to the ability to bind metal ions and other compounds, these DNAzymes can be used to detect Ag^+^, Cu^2+^, Pb^2+^, Hg^2+^, and Sr^2+^ ions [23,24,25,26,27]. This review is thus aimed at summarizing the facts about G-quadruplexes as sensing probes for determining of biologically active compounds.

## 2. G-Quadruplexes as Detectors

### 2.1. Detection of Metal Ions

#### 2.1.1. Detection of K^+^

G-quadruplexes may serve as detectors for potassium using oligonucleotides forming G-quadruplexes and triphenylmethane fluorescent dye crystal violet (CV). As described by Kong *et al.* [28], a K^+^ detection method is based on the fluorescence difference of some CV/G-quadruplex complexes in the presence of K^+^ or Na^+^, and the fluorescence change with the variation of K^+^ concentration. According to the nature of the fluorescence change of CV as a function of ionic conditions, two K^+^ detection modes were introduced. In the first type, with a decrease in the CV fluorescence, oligonucleotides T_3_TT_3_ (5'-GGGTTTGGGTGGGTTTGGG-3') were used, and the fluorescence of CV decreased with an increasing concentration of K^+^. Conversely, in the second type, where oligonucleotides Hum21 (5'-GGGTTAGGGTTAGGGTTAGGG-3') were used, the CV fluorescence with an increasing concentration of K^+^ increased [28]. Another fluorescent detection method for K^+^ was described by Qin *et al.* [29]. Detection of K^+^ was developed using G-quadruplex DNA (c-Myc), which modulated fluorescence enhancement of tetrakis(diisopropylguanidino) zinc phthalocyanine (Zn-DIGP). With an increasing concentration of K^+^ fluorescence of Zn-DIGP increased. In next detection method, G-quadruplex structure stabilized by K^+^ is able to bind hemin; thus, it is forming DNAzyme, which in turn catalyses hydrogen peroxide mediated oxidation of colourless 3,3',5,5'-tetramethylbenzidine (TMB) to a blue product. Under optimal conditions, the colour change is visible to the naked eye within the concentration range from 2 to 1000 µM [30]. Another detection method for K^+^ used G-quadruplex complex with berberine, the plant alkaloid with broad medical uses [31]. After addition of K^+^ single stranded DNA folded into G-quadruplex and after incubation with berberine formation of berberine-G-quadruplex complex occurred leading to a marked increase in fluorescent signal. In the presence of 800 mM of Na^+^ ions fluorescence of the berberine-G-quadruplex was linearly increasing with an increasing concentration of K^+^ within the range from 0.005 to 1.0 mM [31].

#### 2.1.2. Detection of Ag^+^

**Figure 2:**
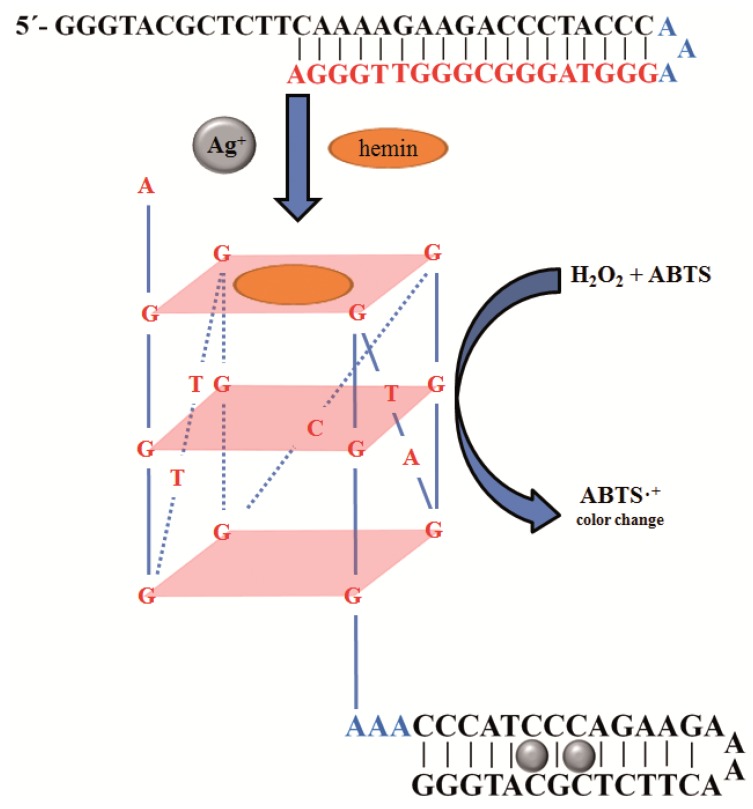
Schematic representation of the G-quadruplex–hemin DNAzyme amplified Ag^+^-sensing method. Adopted and modified according to Zhou *et al.* [25].

A G-quadruplex–hemin DNAzyme-amplified Ag^+^-sensing method was described by Zhou *et al.* [25]. This method is based on the ability of Ag^+^ to stabilize cytosine-cytosine (C–C) mismatches by forming C–Ag^+^–C base pairs. In the absence of Ag^+^, the oligonucleotide strand formed an intramolecular duplex (Figure 2). After addition of Ag^+^ G-rich sequence folds into G-quadruplex structure capable to bind hemin to form a catalytically active G-quadruplex-hemin DNAzyme [25]. In the aforementioned method one oligonucleotide was used. On the other hand, Kong *et al.* [32] used method based on a similar principle, but using two different length oligonucleotide chains for Ag^+^ detection. Also, they took advantage of strong bond between Ag^+^ and cysteine for the detection of cysteine only. Cysteine broke C-Ag^+^-C bonds leading to reformation of the DNA duplex and reduced catalytic activity of the system.

#### 2.1.3. Detection of Hg^2+^

Li *et al.* [23] described a colorimetric method for highly selective and specific detection of Hg^2+^ using Hg^2+^ modulated G-quadruplex-based DNAzyme. Mercury ion (Hg^2+^) is able to specifically bind to the thymine-thymine (T-T) mismatch in a DNA duplex. G-quadruplex DNAs are able to bind hemin to form the peroxidase-like DNAzymes in the folded state. Upon addition of Hg^2+^, the proper folding of G-quadruplex DNAs is inhibited due to the formation of T-Hg^2+^-T complex. This is reflected by the notable change of the Soret band of hemin when investigated by using UV-VIS absorption spectroscopy. As a result of Hg^2+^ inhibition, a sharp decrease in the peroxidase like activity which causes the H_2_O_2_-mediated oxidation of 2,2'-azino-bis(3-ethylbenzothiazoline-6-sulfonic acid)-diammonium salt (ABTS) is observed, accompanied by a change in solution colour [23]. The same principle was used in the work of Jia *et al.* [33], who used the detected mercury further for the detection of cysteine.

#### 2.1.4. Detection of Cu^2+^

An effective G-quadruplex-based probe was constructed by Zhang *et al.* [27] for rapid and sensitive detection of Cu^2+^. In this probe, an anionic porphyrin, protoporphyrin IX (PPIX) served as a reference signal, which binds to G-quadruplex specifically and the fluorescence intensity increases sharply. On the other hand, in the presence of Cu^2+^, the G-quadruplex can catalyse the Cu^2+^ insertion into the protoporphyrin, and the fluorescent intensity is decreased. The assay was shown to be highly specific [27].

#### 2.1.5. Detection of Pb^2+^

**Figure 3:**
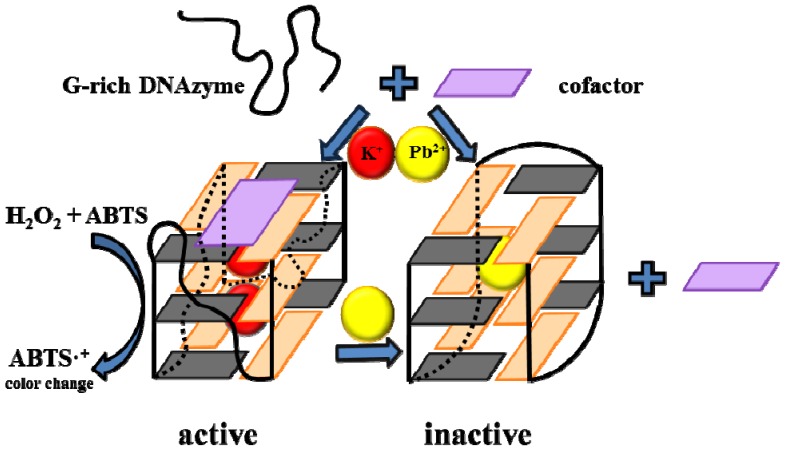
Construction of an INHIBIT logic gate based on the G-rich DNAzyme PW17, with K^+^ and Pb^2+^ as two inputs and absorbance as an output. Cofactor is hemin. In the quadruplex structures, anti and syn guanines are coloured cyan and orange, respectively. Adopted and modified according to Li *et al.* [34].

Lead ions (Pb^2+^) induce a conformational change of the potassium stabilized G-quadruplex DNAzyme and inhibit the peroxidase-like activity. Li *et al.* and Wang *et al.* [34,35] used G-quadruplex as DNAzyme for colorimetric detection of Pb^2+^. G-quadruplex/hemin DNAzyme catalyses hydrogen peroxide mediated oxidation of ABTS, which results in a colour change (Figure 3). After the addition of Pb^2+^ potassium stabilized G-quadruplex/hemin is converted to Pb^2+^-stabilized structure with higher stability but lower DNAzyme activity, which is reflected by an increase in DNA melting temperature on one side but also in a sharp decrease in readout signal on the other side. This allows utilizing this G-quadruplex for quantitative analysis of aqueous Pb^2+^ using the ABTS-H_2_O_2_ colorimetric system. Also luminol-H_2_O_2_ chemiluminescence system was used for Pb^2+^ detection [36]. Using UV/VIS detection, Pb^2+^ was detected at a level of 32 nM, whereas the detection limit using chemiluminescence method was even below 1 nM.

Pb^2+^ could be further detected by fluorescence using G-quadruplex-DNAzyme. The method is based on Pb^2+^ induced increase in DNAzyme activity of oligonucleotide AGRO100 in the presence of hemin, which acts as a cofactor for catalysing of H_2_O_2_-mediated oxidation of the fluorescent dye Amplex^®^ UltraRed (AUR). AGRO100/AUR probe showed high selectivity for the Pb^2+^ ions in comparison with other metal ions. Intensity of AUR fluorescence was proportional to concentration of Pb^2+^ ions within the interval from 0 to 1000 nM [37]. Another type of fluorescent biosensor for Pb^2+^ was designed based on Pb^2+^-induced allosteric quadruplex. In the presence of K^+^ N-methylmesoporphyrin IX (NMM) binds to K^+^ stabilized G-quadruplex, resulting in high fluorescence. After addition of Pb^2+^ binding of Pb^2+^ to G-quadruplex occurs and thereby prevents binding of NMM. This results in a decrease in fluorescence [38]. Graphene oxides (GO) mixed with aptamer-functionalized CdSe/ZnS quantum dots (QDs) can serve as a fluorescent sensor for Pb^2+^. Aptamer-conjugated QDs bind to GO and form GO/QDs-aptamer complex, which allows energy transfer from QDs on GO and fluorescence quenching of QDs. The presence of GO Pb^2+^ induces a conformational change of aptamer to the G-quadruplex, to which GO Pb^2+^ binds. QDs are thus separated from the GO and an increase in fluorescence of QDs occurs [39].

#### 2.1.6. Detection of Ca^2+^

A parallel G-quadruplex-selective iridium (III) complex was developed as a luminiscent probe for G-quadruplex-based detection assay for Ca^2+^ ions in aqueous solution. In this assay, guanine-rich oligonucleotides initially exist in an antiparallel G-quadruplex conformation, resulting in a low luminiscence signal. Upon incubation with Ca^2+^ ions, there is a change of the antiparallel G-quadruplex to a parallel G-quadruplex conformation, which greatly enhances the luminiscence emission of the iridium (III) probe [40].

#### 2.1.7. Detection of Sr^2+^

The inhalation of strontium can cause severe respiratory difficulties, anaphylactic reaction and extreme tachycardia. Strontium can replace calcium in organism, inhibit normal calcium absorption and induce strontium “rickets” in childhood. For detection of strontium ions (Sr^2+^) a simple method using thiazoleorange (TO) on the basis of Sr^2+^ induced conformational change of telomeric DNA in the presence of single-wall nanotubes (SWNTs) was suggested. The limit of detection was 10 nM Sr^2+^[26].

### 2.2. Detection of Anions

#### Detection of I^−^

Li *et al.* [41] developed a simple and sensitive chemiluminescence assay for iodide (I^−^), which is based on iodide extracting Hg^2+^ from DNA featuring a stem-loop structure containing T-Hg^2+^-T. Because the binding of Hg^2+^ and I^−^ is much stronger than that of Hg^2+^ and thymine, I^−^ could extract Hg^2+^ from the stem-loop structure, releasing the DNA, which then bound with K^+^ and transformed into a K^+^ stabilized G-quadruplex (with hemin as a cofactor), which catalyses H_2_O_2_-mediated oxidation of luminol. The produced chemiluminescence as a sensing signal was applied to sensitively and selectively detect iodide with a detection limit of 12 nM.

### 2.3. Detection of Organic Molecules

#### 2.3.1. Amino Acid Detection

**Figure 4:**
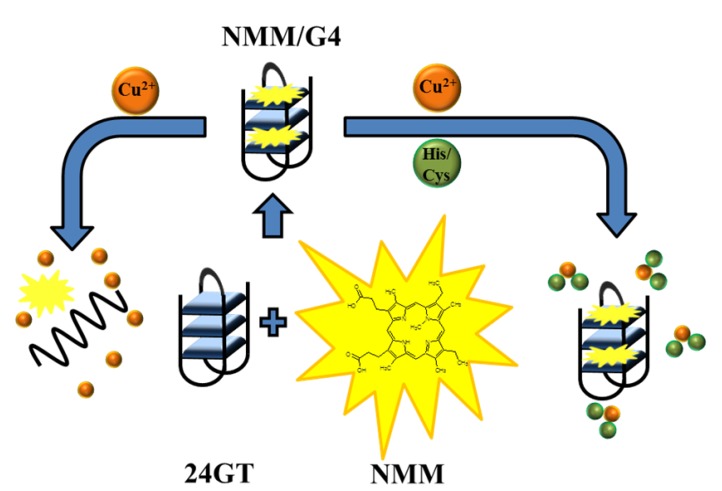
Schematic illustration of the fluorescence change of the NMM/G-4 ensemble under different conditions. The combination of NMM and an intramolecular G-quadruplex generated from 24GT oligonucleotide functions as a signal indicator NMM/G-4 with strong fluorescent intensity. Cupric ion can quench the fluorescence of NMM/G-4 through its coordination with NMM as well as the unfolding of G-quadruplex by Cu^2+^ (as shown in the left side). However, the presence of histidine or cysteine can disturb the interaction between Cu and NMM/G-4 complex due to their interaction with Cu^2+^, generating a distinct fluorescence response from that of cupric ion alone (as shown in the right side). Adopted and modified according to Li *et al.* [43].

Histidine and cysteine detection is critically important, because their abnormal levels are an indicator for many diseases. Li *et al.* [42] demonstrated a quadruplex-based method for detection of histidine and cysteine. The method is based on a highly specific interaction among amino acids (histidine or cysteine), Cu^2+^ and N-methylmesoporphyrin IX in complex with quadruplex (NMM/G-4). The fluorescence intensity of NMM is significantly increased and in the presence of G-quadruplex can be quenched by Cu^2+^. The presence of histidine or cysteine then disturbs the interaction between Cu^2+^ and NMM/G-4 because of the strong binding affinity of Cu^2+^ to the imidazole group of histidine or the interaction of Cu^2+^ with thiol groups of cysteine, leading to distinct fluorescence emission intensity (Figure 4). High selectivity is conferred by the use of cysteine-masking agent N-ethylmaleimide (NEM), which helps to discriminate histidine from cysteine [42].

Cysteine (cys) can be also determined together with glutathione (GSH) by the quadruplex-based method using Hg^2+^ and NMM. The system consists of two single stranded DNA (ssDNA) with thymine-thymine (T-T) mismatches and used Hg^2+^ as a mediator, and NMM as the signal reporter. In the absence of analyte (cys or GSH) two ssDNA containing T-T mismatches react with Hg^2+^ to form T-Hg^2+^-T dsDNA structure in the solution, which hampers the formation of a G-quadruplex structure. However, in the presence of GSH or cys the analyte reacts with Hg^2+^ to keep DNA probes in a free single state, resulting in the effective formation of a G-quadruplex structure of the DNA probe. Subsequently, due to the strong interaction between G-quadruplex structure and NMM, fluorescence is greatly enhanced. This method exhibited a linear relationship between peak fluorescence intensity and concentration of GSH in the range of 10–400 nM with a limit of detection (LOD) of 9.6 nM. A linear range for Cys detection was obtained in the concentration range of 10–500 nM with an LOD of 10 nM [44]. Another sensitive method for detection of cysteine was described by Su *et al.* [45]. The mechanism is based on the oxidation of cysteine by H_2_O_2_, which prevents the catalysis of the ABTS-H_2_O_2_ reaction by G-quadruplex halves. With the addition of Cys, the amount of the blue-green-coloured free radical cation ABTS^+^ was reduced and there was a significant decrease in an absorbance. The concentration of cysteine was determined by UV-VIS spectroscopy and colour change was already discernible to the naked eye. The calibration curve showed that the net absorption value at 421 nm linearly increased over the Cys concentration range of 0.005–100 µM with a detection limit of 5 nM.

#### 2.3.2. Glucose Detection

Colorimetric method for glucose detection in urine was developed. Oxidation of glucose is converted to colour change of 10-acetyl-3,7-dihydroxy phenoxazine (ADHP) using DNAzyme, which consist of G-quadruplex and hemin. DNAzyme catalyses oxidation of colourless ADHP to red resorufin by H_2_O_2_, which is product of glucose oxidase catalysed reaction of glucose and oxygen. Oxidation of glucose (colour change of ADHP) is the result of these reactions [46].

#### 2.3.3. Cholesterol Detection

G-quadruplex/hemin complex can be also used for colorimetric detection of cholesterol. By this DNAzyme catalysed oxidation of colourless ABTS^2−^ by H_2_O_2_ to colourful ABTS^−^. In this case, H_2_O_2_ is produced by a reaction of cholesterol and oxygen catalysed by cholesterol oxidase. Colour change of ABTS^2−^ is the consequence of cholesterol oxidation [47].

#### 2.3.4. ATP Detection

Adenosine triphosphate (ATP) can be also detected by DNAzyme aptamer sensor, which uses two DNA sequences. First functional chain (A chain) consists of two parts—anti-ATP aptamer (recognition part) and DNAzyme (signal transduction part). Second sequence is used as a blocking chain (B chain), which can hybridize with A chain. After addition of hemin and ATP, hybridized chains unfold. DNAzyme in functional chain is creating G-quadruplex with hemin, thus it catalyses oxidation of ABTS by H_2_O_2_[48]. In another work, aptamers immobilised on the electrode surface can be used for ATP detection. One aptamer serves as recognition element of ATP and second as a signal source. First probe L1 contains ATP aptamer and a part of hemin aptamer and second complementary chain to ATP aptamer and the rest of hemin aptamer. L1 was immobilised on the electrode surface. L2 hybridised with L1 and L1-L2 complex was formed, thus two parts of hemin aptamer became closer. Hemin formation and L1-L2 chain results in G-quadruplex. Hemin immobilised inside quadruplex produced strong electrochemical signal. When ATP is added, duplex is disrupted and L2 is released to solution. Hemin is not captured and no signal is detected [49].

#### 2.3.5. Detection of Cocaine

For detection of cocaine DNAzyme based colorimetric method in combination with the magnetic nanoparticles was described. Cocaine aptamer fragment SH-C2 was covalently bound to the magnetic nanoparticles. Target cocaine and another cocaine aptamer fragment (C1) are instilled into the G-rich strand (C1-AG4). With this region SH-C2 bound on magnetic nanoparticles hybridizes. C1-AG4 can form with hemin DNAzyme catalysing hydrogen peroxide-mediated oxidation of 3,3,5,5-tetramethylbenzidine sulphate (TMB), which leads to colour change of the solution. Using magnetic nanoparticles as separation and amplification elements background signal and the interference of real samples can be effectively reduced [50].

### 2.4. Detection of Nucleic Acids

#### 2.4.1. MicroRNA Detection

**Figure 5:**
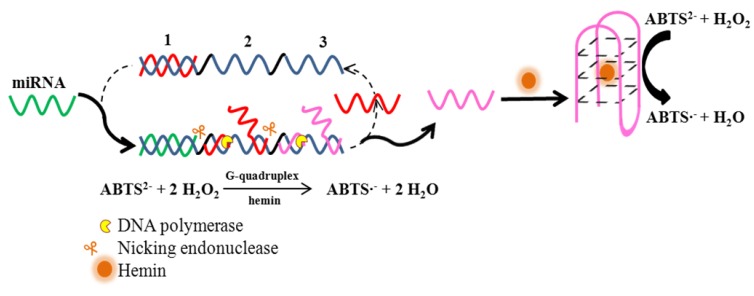
Schematic illustration of miRNA detection based on isothermal exponential amplification-assisted generation of catalytic G-quadruplex DNAzyme. Below: the reaction schemes of ABTS^2−^ catalysed by G-quadruplex DNAzyme in the presence of hemin and H_2_O_2_. Adopted and modified according to Wang *et al.* [51].

A sensitive method for microRNAs detection was also suggested. It is based on isothermal exponential amplification via formation of G-quadruplex DNAzymes with catalytic activity. This method involves an elongation of DNA chain by DNA polymerase, single-strand nicking and a catalytic reaction of G-quadruplex/hemin complex. The target miRNA initiates repeating synthesis and nicking of two oligonucleotide fragments and substitution fragments by thermostabile polymerase and nicking endonuclease. One oligonucleotide fragment has the same sequence as the target miRNA, except that deoxyribonucleotides and thymine are replaced ribonucleotides and uridine in the miRNA, to activate new cyclic chain reactions of polymerization, nicking and displacement reactions as the target miRNA (Figure 5). Another way is the signal molecule of horseradish peroxidase (HRP)-mimicking G-quadruplex DNAzyme. With such designed signal amplification processes, the suggested assay showed a quantitative analysis of sequence-specific miRNAs in a wide range from 1 fM to 100 nM with a low detection limit of 1 fM. Moreover, this assay demonstrated excellent differentiation ability for the mismatch miRNAs targets and good performance in biological samples [51].

#### 2.4.2. Detection of p53 Gene Sequence

DNAzymes can serve as a p53 DNA sequence sensors. This sensing system is based on DNAzyme molecular beacon (MBzyme), where G-rich segment (anti-hemin aptamer) capable of DNAzyme creation is introduced into the p53 recognition probe. Stable hairpin, which consists of primer, recognition sequence and anti-hemin aptamer, is locking DNAzyme active centre and is not able to exhibit peroxidase activity. Hybridization between target p53 gene sequence and recognition probe is responsible for hairpin disruption and G-quadruplex creation. G-quadruplex binds strongly hemin and creates DNAzyme, which is able to catalyse H_2_O_2_-mediated oxidation of ABTS. When hairpin is disrupted and G-quadruplex is created, Klenow Fragment exo^−^ (KF) can be used to cleave out hybridized p53 sequence from recognition probe and in the presence of deoxyribonucleotides can also synthesize complementary chain to recognition probe. Released p53 sequence can disrupt other hairpin. This unique strategy of strand-displacement amplification leads to generation of multiple numbers of active DNAzymes and enhances detection sensitivity [43].

#### 2.4.3. Detection of Gene Deletion

He *et al.* [52] developed a G-quadruplex-based switch-on luminescence assay for the detection of gene deletion using a cyclometallated iridium(III) complex as a G-quadruplex-selective probe. Upon hybridization with the target DNA, the two split G-quadruplex-forming sequences assemble into a split G-quadruplex, which greatly enhances the luminescence emission of the iridium(III) probe. The assay is simple and highly selective.

#### 2.4.4. Detection of Genetically Modified Organisms

G-quadruplexes can be also used for detection of genetically modified organisms (GMOs). As it is known that the cauliflower mosaic virus (CaMV) 35S promoter is widely used in most transgenic plants, Qiu *et al.* [53] designed a simple method based on the detection of a section target DNA (DNA-T) from the transgene CaMV 35S promoter. In this method, the full-length guanine-rich single-strand sequences were split into fragments (Probe 1 and 2); each part of the fragment possesses two GGG repeats. In the presence of K^+^ ion and berberine, if a complementary target DNA of the CaMV 35S promoter was introduced to hybridize with Probe 1 and 2, a G-quadruplex-berberine complex was formed and generated a strong fluorescence signal. The generation of fluorescence signal indicated the presence of CaMV 35S promoter. This method is therefore able to identify and quantify GMOs [53].

### 2.5. Detection of Proteins

#### 2.5.1. Detection of Neutrophil Elastase

G-quadruplex luminescent iridium (III) complex was designed for sensitive and selective detection of human neutrophil elastase (HNE) in a homogeneous solution. HNE aptamer was initially hybridized with the complementary DNA strand and weak bond iridium (III) complex to the DNA duplex showed low luminescence signal. Due to the very high affinity for HNE aptamer to HNE immediately after the addition of HNE causes separation of duplex structures and promotes the formation of HNE-protein aptamer. G-quadruplex formed from HNE binding aptamer interacts with iridium (III) causing enhanced luminescence signal [54].

#### 2.5.2. RNAse H Detection

RNAse H is ribonuclease, which can specifically degrade RNA chain in RNA-DNA duplex by endonucleolytic mechanism. Fluorescence method based on the use of G-quadruplexes was developed for sensitive and easy-to-perform detection of activated or inhibited state of RNAse H. Guanine rich regions in DNA, which are released after cleavage of the RNA chain by RNAse H, are folded in the presence of monovalent ions and form quadruplexes. These G-quadruplexes interact specifically with N-methyl mesoporphyrin IX (NMM) and cause significant increase in fluorescence, which is used as a reporter reaction. This new method is simpler, faster, more comfortable and more promising than other methods [55].

#### 2.5.3. Thrombin Detection

DNAzyme based detection can be used for quantification of thrombin, too. Thrombin-binding aptamer (TBA) binds hemin and creates catalytic complex. Its catalytic activity is increased by addition of thrombin and enables highly specific and sensitive colorimetric detection of thrombin. Detection mechanism is based on oxidation of colourless ABTS^2−^ by H_2_O_2_, which results in colourful ABTS^−^ [56]. Thrombin was also detected by Li *et al.* [57]. The authors came out from the assumption that thrombin has two binding sites and thus it can create sandwich with two aptamers. First aptamer is immobilized on gold substrate to capture target protein. Second aptamer connected to DNAzyme can be bound to second binding site, so protein can be detected by luminal-H_2_O_2_ method. This interesting method can be also used to detect other proteins, which have two binding sites for aptamer. Thrombin can be also detected with use of exonuclease I and signal amplification by direct electron transfer (DET) of hemin. If thrombin is missing, TBA situated on an electrode surface is digested by exonuclease I, which avoids the association of hemin and significantly minimizes the background noise. The presence of hemin supports a formation of G-quadruplex from TBA and prevents it from degrading by exonuclease. Hemin bound to G-quadruplex amplifies the signal [58].

#### 2.5.4. Detection of HIV-1 Integrase and Nucleoline

Two G-quadruplex aptamers AGRO100 and T30695 were described as multifunctional aptamers binding protein ligands nucleoline or HIV-1 integrase and hemin. They can form DNA/hemin complex exhibiting peroxidase activity, which may be used for the sensitive detection of proteins. This is illustrated in an application of AGRO100 for chemiluminescent detection of nucleoline expressed on the surface of HeLa cells. Nucleoline is marked with DNAzyme/hemin/AGRO100 and determined with luminol H_2_O_2_ system. AGRO100 acts as anticancer aptamer while T30695 aptamer as anti-HIV aptamer [59].

#### 2.5.5. Detection of DNA Polymerase Proofreading Activity

DNA repair processes are responsible for upholding the integrity and stability of genomic DNA. Consequently, the development of analytical assays to monitor enzymes involved with DNA repair pathways is of great interest in a variety of disciplines, including biochemistry, cell biology, and biotechnology. Leung *et al.* [41] reported a luminescent switch-on label-free G-quadruplex-based assay for the rapid and sensitive detection of DNA polymerase 3'–5' proofreading activity using a novel iridium(III) complex as a G-quadruplex-selective probe. The interaction of the iridium(III) complex with the G-quadruplex motif facilitates the highly sensitive switch-on detection of polymerase proofreading activity. Using T4 DNA polymerase as a model enzyme, the assay achieved high sensitivity and selectivity for T4 DNA polymerase.

### 2.6. Detection of Other Analytes

#### 2.6.1. Cisplatin Detection

DNAzymes may serve as an electrochemical biosensor for detection of cisplatin. Hemin/G-quadruplex DNAzyme wires (supersandwich DNAzyme structure) containing many units of hemin/G-quadruplex DNAzyme were used as electrochemical signal amplifiers. Supersandwich DNAzyme exhibits peroxidase activity and is capable of reducing hydrogen peroxide. After the addition of cisplatin to this complex there were changes in the structure and catalytic effect on hydrogen peroxide was affected. With an increasing concentration of cisplatin gradually conformational changes of DNAzyme occurred and its catalytic effect on H_2_O_2_ was reduced, which is the principle of cisplatin detection. Linear relationship between the concentration of cisplatin and obtained electrochemical signal was also determined [60].

#### 2.6.2. Detection of Antioxidants

With DNAzymes antioxidant activity and its influence on quenching free radicals can be colorimetrically monitored. DNAzyme catalyses oxidation of colourless ABTS by H_2_O_2_ to colour radical ABTS^+^ that can be quenched by antioxidants, which is as a result reflected as a colour change. This method can be effectively used for the quantitative determination of the concentration of antioxidants and to evaluate the antioxidant capacity of various antioxidants and real samples [24]. Modified screening method for detection of antioxidants was also developed [61]. This method uses the ABTS^+^ radical formed by ABTS–H_2_O_2_ system, which is catalysed by G-quadruplex and stabilized with adenosine triphosphate (ATP). In the presence of ATP only, the life of the radical cation was prolonged six times. Due to this fact the antioxidant activity of real samples can be easily determined and compared. The result can be detected by the naked eye, too [61].

### 2.7. Performance Characteristics of the G-Quadruplex Based Detection Methods

In Table 1, a list of certain performance characteristics of the detection methods based on the G-quadruplex can be seen. The first part of the table shows the detection methods for metal ions. Detection limits range from 1 nM in luminescence detection of Pb^2+^ to 2 µM for colorimetric detection of K^+^. Working ranges for certain colorimetric methods achieve 10–600 nM (Ag^+^, Hg^2+^); larger working ranges at colorimetric methods may be achieved: 50–2,500 nM (Hg^2+^), 100–3,000 nM (Ag^+^), 32–60,000 nM (Pb^2+^), and 2–1,000 µM (K^+^). Similar working ranges can be achieved also for fluorescence and luminescence methods (K^+^, Cu^2+^, Pb^2+^, and Sr^2+^). Selectivity for the determination of metals was tested by adding from 5 to 12 different metal cations to the reaction mixture. Methods for detection of metals are highly selective. Next, Table 1 shows examples of colorimetric detection of cysteine and histidine fluorescence detection. The working range at the detection of histidine ranges from 3 to 15,000 nM and at detection of cysteine is greater than 5–100,000 nM. Selectivity was tested using 19 amino acids. The electrochemical detection of cisplatin was working in the range of 50–5,000 nM. At the detection of glucose and cholesterol, the working ranges were 3–100 µM and 1–30 µM, respectively. The lowest detection limits were achieved for colorimetric detection of micro RNA and DNA (1 fM and 25 fM, respectively).

**Table 1:** Overview of some performance parameters (sensitivity, working range, selectivity) of G-quadruplex based detection methods.

Analyte	Type of Detection, Indicator	Detection Limit	Working Range	Selectivity Tested in the Presence of	Reference
K^+^	Fluorescence CV	1 mM	1–15 mM	Na^+^, Mg^2+^, Ca^2+^	[28]
K^+^	Fluorescence Zn-DIGP	0.8 µM	0.8–400 µM	Li^+^, NH_4_^+^, Na^+^, Mg^2+^, Zn^2+^, Ca^2+^, Cu^2+^, Fe^3+^	[29]
K^+^	Colorimetric TMB	2 µM	2–1,000 µM	Li^+^, NH_4_^+^, Na^+^, Mg^2+^, Ca^2+^, Cs^2+^	[30]
K^+^	Fluorescence Berberine	2 µM	5–1,000 µM	Na^+^, Mg^2+^, Ca^2+^	[31]
Ag^+^	Colorimetric ABTS	6.3 nM	5–600 nM	Ca^2+^, Mg^2+^, Cu^2+^, Mn^2+^, Zn^2+^, Co^2+^, Cd^2+^, Pb^2+^, Hg^2+^, Ni^2+^, Fe^3+^, Cr^3+^	[25]
Ag^+^	Colorimetric ABTS	64 nM	100–3,000 nM	Ca^2+^, Mg^2+^, Cu^2+^, Mn^2+^, Zn^2+^, Co^2+^, Cd^2+^, Pb^2+^, Hg^2+^, Ni^2+^, Fe^3+^, Cr^3+^	[62]
Hg^2+^	Colorimetric ABTS	50 nM	50–2,500 nM	Ca^2+^, Mg^2+^, Cu^2+^, Zn^2+^, Cd^2+^, Pb^2+^, Fe^2+^, Fe^3+^, Cr^3+^	[23]
Hg^2+^	Colorimetric ABTS	9.2 nM	10–600 nM	Ca^2+^, Mn^2+^, Cu^2+^, Zn^2+^, Cd^2+^, Co^2+^, Pb^2+^, Ni^2+^, Fe^3+^, Cr^3+^	[33]
Cu^2+^	Fluorescence G-quadruplex–PPIX	3 nM	8–2,000 nM	Ca^2+^, Mn^2+^, Mg^2+^, Zn^2+^, Cd^2+^, Co^2+^, Pb^2+^, Hg^2+^, Ni^2+^, Fe^2+^, Fe^3+^, Cr^3+^	[27]
Pb^2+^	Colorimetric ABTS	32 nM	32–60,000 nM	Ca^2+^, Cu^2+^, Mg^2+^, Zn^2+^, Cd^2+^, Hg^2+^, Fe^3+^	[36]
Pb^2+^	Luminescence Luminol	1 nM	1–10,000 nM	Ca^2+^, Cu^2+^, Mg^2+^, Zn^2+^, Cd^2+^, Hg^2+^, Fe^3+^	[36]
Sr^2+^	Luminiscence Iridium(III) complex	13 nM	13–20,000 nM	K^+^, Li^+^, Na^+^, Ba^2+^, Ni^2+^, Ca^2+^, Zn^2+^, Mg^2+^, La^3+^, Cr^3+^, Al^3+^, Ti^3+^	[63]
Cysteine	Colorimetric ABTS	5 nM	5–100,000 nM	Ala, Arg, Asp, Gln, Glu, His, Ile, Gly, Asn, Leu, Lys, Met, Phe, Pro, Ser, Thr, Trp, Tyr, Val	[45]
Histidine	Fluorescence NMM, Cu^2+^	3 nM	3–15,000 nM	Ala, Arg, Asp, Cys, Gln, Glu, Ile, Gly, Asn, Leu, Lys, Met, Phe, Pro, Ser, Thr, Trp, Tyr, Val	[42]
Cisplatin	Electrochemical CV	20 nM	50–5,000 nM	Transplatin	[60]
Micro RNA 141	Colorimetric ABTS	1 fM	1 fM–100 nM	miR-429, miR-200b, let-7d, miR-21	[51]
p53 DNA	Colorimetric ABTS	25 fM	25 fM–500 nM	2 partly complementary target p53 DNA	[43]
Glucose	Colorimetric ADHP	1 µM	3–100 µM	Acetaminophen, glycerin, serine, uric acid, ascorbic acid	[46]
Cholesterol	Colorimetric ABTS	0.1 µM	1–30 µM	Phenol, ascorbic acid, glycerin, glucose, uric acid, serine, cholesterol ester	[47]

## 3. G-Quadruplexes and Nanoparticles

G-quadruplex-stabilizing compounds (ligands) have become a focus of attention recently, as they may interfere with the telomere structure, telomere elongation/replication, and proliferation of cancer cells. Chen *et al.* [64] described a system for screening of G-quadruplex ligands using gold nanoparticles (AuNPs). This method is based on the fact that guanine rich DNA modified with AuNPs is stable at certain salt concentrations and unmodified AuNPs tend to aggregate together. In the presence of G-quadruplex binding ligand DNA stuck on the gold nanoparticles changes conformation from linear to G-quadruplex, which protects DNA from enzymatic degradation. On the other hand, DNA in the absence of G-quadruplex binding ligand may be easily enzymatically cleaved, which leads to the change of colour from red to violet. Using gold nanoparticles, even low concentrations of G-quadruplex DNA can be detected. Gold nanoparticles may together with G-quadruplex DNA serve as detectors of various G-quadruplex ligands [65].

In another work Zhou *et al.* [66] described the electrochemical detection of miRNAs using gold nanoparticles (AuNPs). Hairpin DNA probe was immobilized on the electrode surface modified by AuNPs with a segment at the 3'-end complementary to the miRNA-21 segment and a segment at the 5'-end for capture DNA. After hybridization with the target miRNA hairpin structure was spread and was further hybridized with the capture DNA on AuNPs. AuNPs contained two types of DNA, one complementary to the hairpin structure of the DNA probes, while the other served as an aptamer for hemin. The electrochemical signal of hemin located in the centre of G-quadruplex was measured using chronoamperometry. Target miRNA-21 was analysed with a detection limit of 3.96 pM.

Liang *et al.* [67] described a method for detection of the aforementioned thrombin using aptamer modified gold-rhenium (AuRe) nanoprobe in combination with a resonance scattering (RS) spectral method. In the presence of alkali metal ions (K^+^, Na^+^) a change of the aptamer conformation on AuRe nanoparticles to G-quadruplex structure occurs. In the absence of thrombin resonance scattering signal is very weak. Conversely, in the presence of thrombin, the AuRe/aptamer (G-quadruplex)/thrombin complex is formed, which exhibits RS peak at 560 nm. As the amount of thrombin increased, the amount of AuRe-aptamer-thrombin cluster also increased, and the size of cluster became large. This clusters or more precisely AuRe particles in clusters greatly enhance the scattering signal. AuRe nanoparticles efficiently scatter light as a consequence of resonance between the incident photon and the interface electron on the nanoparticle surface. Gold nanorods (AuNR) may serve as a detector for the human telomeric DNA hybridization and formation of G-quadruplexes [68].

Gold nanorods (AuNRs) as colorimetric probe were used for the rapid detection of Pb^2+^. The method is based on a conformational change in the transition from single-stranded DNA to G-quadruplex. Electrostatic interactions between the DNA probe and AuNRs induce spatial closure of AuNRs. In the presence of Pb^2+^ G-quadruplex increases charge density around the DNA, which has the effect of reinforcing the electrostatic interaction between AuNRs and DNA. This led to a reduction in longitudinal absorption of AuNRs because of stronger interaction caused aggregation of AuNRs. The decrease in the longitudinal absorption is directly proportional to the concentration of Pb^2+^ [69]. In the work of Chen *et al.* potassium ions (K^+^) were detected by gold nanoparticles (AuNPs). To assay for K^+^ ions, the thiolated aptamers were conjugated to AuNPs separately via the strong Au-S bond. In the absence of K^+^, the aptamer-modified AuNPs dispersed well in the solution, and the G-rich nucleic acid was in the random coil state. However, once a solution containing K^+^ was introduced, K^+^ could specifically bind to the aptamer and induced the aptamer-AuNPs switching from a well dispersed state to an aggregated one, resulting in a change in the UV-vis absorption spectra of the solution. The linear range of the colorimetric aptasensor covered a large variation of K^+^ concentration from 5 nM to 1 μM and the detection limit of 5 nM was obtained. Moreover, this assay was able to detect K^+^ with high selectivity and had great potential applications [70].

## 4. Conclusions

Review focuses on the use of G-quadruplexes as detectors of heavy metals. Besides heavy metals, other analytes using G-quadruplexes can be analysed such as organic molecules, nucleic acids, and proteins. An important screening technique for G-quadruplex ligands is the use of G-quadruplexes in combination with the gold nanoparticles. The basis of detections using G-quadruplexes is DNA conformational change induced by the presence of analyte, resulting in a decrease or increase in peroxidase activity, fluorescence, or electrochemical signal of the used probe. In comparison with other detection methods, the detection using G-quadruplexes is simpler, faster, more sensitive, and less expensive, without expensive instruments and fluorescently labelled oligonucleotides. Moreover, the compound of interest can be detected at very low concentrations. In most cases, the detection is possible by naked eye.
